# Efficacy of a vaginal suppository formulation prepared with *Acacia arabica* (Lam.) Willd. gum and *Cinnamomum camphora* (L.) J. Presl. in heavy menstrual bleeding analyzed using a machine learning technique

**DOI:** 10.3389/fphar.2024.1331622

**Published:** 2024-02-12

**Authors:** Mohamed Joonus Aynul Fazmiya, Arshiya Sultana, Md Belal Bin Heyat, Saba Parveen, Khaleequr Rahman, Faijan Akhtar, Azmat Ali Khan, Amer M. Alanazi, Zaheer Ahmed, Isabel de la Torre Díez, Julién Brito Ballester, Tirumala Santhosh Kumar Saripalli

**Affiliations:** ^1^ Department of Ilmul Qabalat wa Amraze Niswan, National Institute of Unani Medicine, Ministry of AYUSH, Bengaluru, India; ^2^ CenBRAIN Neurotech Center of Excellence, School of Engineering, Westlake University, Hangzhou, China; ^3^ College of Electronics and Information Engineering, Shenzhen University, Shenzhen, China; ^4^ Department of Ilmul Saidla, National Institute of Unani Medicine, Ministry of AYUSH, Bengaluru, India; ^5^ School of Computer Science and Engineering, University of Electronic Science and Technology of China, Chengdu, China; ^6^ Pharmaceutical Biotechnology Laboratory, Department of Pharmaceutical Chemistry, College of Pharmacy, King Saud University, Riyadh, Saudi Arabia; ^7^ Central Council for Research in Unani Medicine, New Delhi, India; ^8^ Department of Signal Theory and Communications, University of Valladolid, Valladolid, Spain; ^9^ Research Group on Foods, Nutritional Biochemistry and Health, Universidad Europea del Atlántico, Santander, Spain; ^10^ Research Group on Foods, Nutritional Biochemistry and Health, Universidad Internacional Iberoamericana, Arecibo, PR, United States; ^11^ Research Group on Foods, Nutritional Biochemistry and Health, Universidad de La Romana, La Romana, Dominican Republic; ^12^ Regional Research Institute of Unani Medicine, Chennai, India

**Keywords:** female disorder, heavy menstrual bleeding, Unani system of medicine, drug design, artificial intelligence with botanical drug, medical intelligence, *Acacia arabica* gum, *Cinnamomum camphora*

## Abstract

**Objective:** This study aims to determine the efficacy of the *Acacia arabica* (Lam.) Willd. and *Cinnamomum camphora* (L.) J. Presl. vaginal suppository in addressing heavy menstrual bleeding (HMB) and their impact on participants' health-related quality of life (HRQoL) analyzed using machine learning algorithms.

**Method:** A total of 62 participants were enrolled in a double-dummy, single-center study. They were randomly assigned to either the suppository group (SG), receiving a formulation prepared with *Acacia arabica* gum (*Gond Babul*) and camphor from *Cinnamomum camphora* (*Kafoor*) through two vaginal suppositories (each weighing 3,500 mg) for 7 days at bedtime along with oral placebo capsules, or the tranexamic group (TG), receiving oral tranexamic acid (500 mg) twice a day for 5 days and two placebo vaginal suppositories during menstruation at bedtime for three consecutive menstrual cycles. The primary outcome was the pictorial blood loss assessment chart (PBLAC) for HMB, and secondary outcomes included hemoglobin level and SF-36 HRQoL questionnaire scores. Additionally, machine learning algorithms such as k-nearest neighbor (KNN), AdaBoost (AB), naive Bayes (NB), and random forest (RF) classifiers were employed for analysis.

**Results:** In the SG and TG, the mean PBLAC score decreased from 635.322 ± 504.23 to 67.70 ± 22.37 and 512.93 ± 283.57 to 97.96 ± 39.25, respectively, at post-intervention (TF3), demonstrating a statistically significant difference (*p* < 0.001). A higher percentage of participants in the SG achieved normal menstrual blood loss compared to the TG (93.5% vs 74.2%). The SG showed a considerable improvement in total SF-36 scores (73.56%) compared to the TG (65.65%), with a statistically significant difference (*p* < 0.001). Additionally, no serious adverse events were reported in either group. Notably, machine learning algorithms, particularly AB and KNN, demonstrated the highest accuracy within cross-validation models for both primary and secondary outcomes.

**Conclusion:** The *A. arabica* and *C. camphora* vaginal suppository is effective, cost-effective, and safe in controlling HMB. This botanical vaginal suppository provides a novel and innovative alternative to traditional interventions, demonstrating promise as an effective management approach for HMB.

## 1 Introduction

Heavy menstrual bleeding (HMB) is diagnosed as excessive or prolonged uterine bleeding per menstruation of more than 80 mL or for 7 days in a normal cycle (Magnay et al., 2020). Approximately 10%–35% of women visit the hospital with complaints of HMB throughout their reproductive years, and 5% seek medical attention to have HMB investigated (Chaplin, 2018). HMB has a substantial influence on a woman’s quality of life (QoL); hence, essential intervention must focus on QoL rather than just blood loss (Azizkhani et al., 2018). According to Unani medicine, HMB is characterized by excessive or prolonged menstrual blood loss during the menstrual cycle (Al-Jurjānī, 2010; Majusi, 2010). Unani scholars posit that the primary cause of HMB is weak retentive power, excessive excretory power, or a combination of both. Additionally, any deviation from normal temperament (*Mizaj*), particularly a hot (*Harr*) and dry (*Yabis*) disposition, is believed to weaken the uterus and its vessels, impacting the uterine excretory power. This, in turn, leads to the opening and dilation of the mouths of uterine vessels, resulting in heavy menstrual flow (Jurjani, 2010; Sina, 2010). Since there is no universally accepted test-and-treat strategy for HMB in women, the first line of defense is typically empiric pharmacological treatment without further research (Azizkhani et al., 2018). Due to the limitations of current medications, the absence of specific treatment strategies, and limited studies on side effects and drug interactions, the full spectrum of complementary and alternative systems has grown immensely and gained popularity (Tansaz et al., 2016). Plant products act through four strategies to regulate irregular uterine bleeding: estrogenic action, inflammatory process inhibition, inhibition of prostaglandin formation, and anti-proliferative effect on human cervical cancer cells (Magnay et al., 2020).

India uniquely possesses its own recognized traditional medicine systems: Ayurveda, Yoga, Unani, Siddha, and Homeopathy (AYUSH) (Ahmad et al., 2021). Unani researchers provide valuable insights into the diagnosis and treatment of menstrual diseases, considering regular menstruation as a crucial indicator of a woman’s health (Sina, 2010; Al-Jurjānī, 2010). Unani medicine offers effective treatments for HMB in various forms, including oral single or compound form and/or topical vaginal pessary, vaginal suppository, sitz bath, lotion, and balm. In Unani texts, a vaginal suppository of *Acacia arabica* (Lam.) Willd. [Fabaceae] gum (Acacia gum) and camphor from *Cinnamomum camphora* (L.) J. Presl. [Lauraceae] is recommended for controlling HMB. Acacia gum (*Gond Babul*) possesses astringent, anti-inflammatory, styptic, and desiccant properties (Farzana and Sultana, 2020) and is known for treating abnormal vaginal discharge and heavy menstrual bleeding. Unani researchers explain that hemostatic drugs typically have a dry and cold temperament, facilitating capillary constriction and surrounding structure support (Tansaz et al., 2016). Camphor (*Kafoor*) possesses ethnomedicinal properties such as astringent (*Qabis*), anti-inflammatory (*Muhallil*) (Khan and Muhammad, 2018), and external anesthetic and analgesic (*Musakhin*) properties (Kabir al-Din, 2007). Acacia gum and camphor contain tannins that contribute to the coagulation of cellular proteins and the constriction of the capillary endothelium (Tansaz et al., 2016). *A. arabica* gum is pharmacologically reported to have powerful astringent, styptic, analgesic, antioxidant, and anti-inflammatory properties (Massiha and Muradov, 2015; Garg and Jain, 2016). *C. camphora* has potent prostaglandin inhibitors that are astringent, hemostatic, and anti-inflammatory (Daffalah and al-Mustafa, 1996; Karashima et al., 2007; Fan et al., 2020; Hussain et al., 2021; Lee et al., 2022). Acacia gum is rich in tannin with bioactive molecules such as ellagic acid, gallic acid, and tannic acid, which have astringent properties (Ali, 2012; Elgailani and Ishak, 2014; Saeedi et al., 2020). Acacia gum’s aqueous extracts contain polymeric content that shortens the activated partial thromboplastin time (aPTT) and prothrombin time (PT), has hemostatic effects, and accelerates blood coagulation (Bhatnagar et al., 2013). *A. arabica* was reported to be safe in a single dose in experimental animal mice (Alli et al., 2015), and camphor was safe in a proper dose in adult humans. The lethal dose of camphor has been reported to be 5 to 20 gm (Nadkarni KM, 2004).

Various Unani formulations such as *Sharbat-i-Anjabar* (Jahan et al., 2016), *Safuf-i-Hābis* (Fathima and Sultana, 2012), *Qurs-i-Hābis* (Mukhtar et al., 2019), *Gulnār* (Goshtasebi et al., 2015), and dry cupping (*Hijama bila Shart*) (Sultana and Rahman, 2012) have been validated and clinically proven for their effectiveness in treating HMB. Few studies on female disorders included machine learning methods in their clinical trials, including pelvic inflammatory disease (Qayyum et al., 2023) and premenstrual syndrome (Sultana et al., 2022b). However, to date, there has been no study assessing the efficacy of a vaginal suppository formulation prepared with *A. arabica* gum and *C. camphora* camphor in addressing HMB (Khan, 2006) using a machine learning model.

This study intended to compare the efficacy of the formulation prepared with acacia gum (*Gond Babul*) and camphor (*Kafoor*) vaginal suppository with tranexamic acid on the pictorial blood loss assessment score (PBLAC), health-related quality of life (HRQoL), and hemoglobin levels in human participants with HMB. The research question was “whether acacia gum and camphor vaginal suppositories would be efficacious to reduce HMB and, thereby, improve the participant’s HRQoL.” Beyond conventional methods, artificial intelligence (AI) (Benifa et al., 2023), particularly machine learning models, was utilized to classify experimental and standard groups. Additionally, this study used experimental data related to heavy menstrual bleeding for the classification of the vaginal suppository group (SG) as an experimental group and tranexamic group (TG) as a standard control group. The present study used four types of machine learning algorithms, namely, k-nearest neighbor (KNN), AdaBoost (AB), naive Bayes (NB), and random forest (RF) (Belal Bin Heyat et al., 2022; Bin Heyat et al., 2022; Pal et al., 2022; Tripathi et al., 2022; Qayyum et al., 2023). It makes significant contributions by proposing an improved alternative treatment for HMB through the utilization of acacia gum (Gond Babul) and camphor (Kafoor) vaginal suppositories. Additionally, it involves the design and development of this innovative botanical drug vaginal suppository with a focus on standardization and purity testing, including microbial loads, heavy metal, and HPTLC fingerprint analysis. This study also explores the application of AI for the classification of experimental data through machine learning models. Moreover, the research delves into understanding the role of oxidative stress, inflammation, and immune responses in abnormal uterine bleeding. It also seeks to elucidate the mechanism of action for the bioactive metabolites and molecules present in camphor and acacia gum, highlighting their potential as anti-inflammatory, antioxidant, and hemostatic agents.

The proposed study is organized in the standard format, including introduction, materials and methods, results, discussion, and conclusion.

## 2 Materials and methods

**FIGURE 1:**
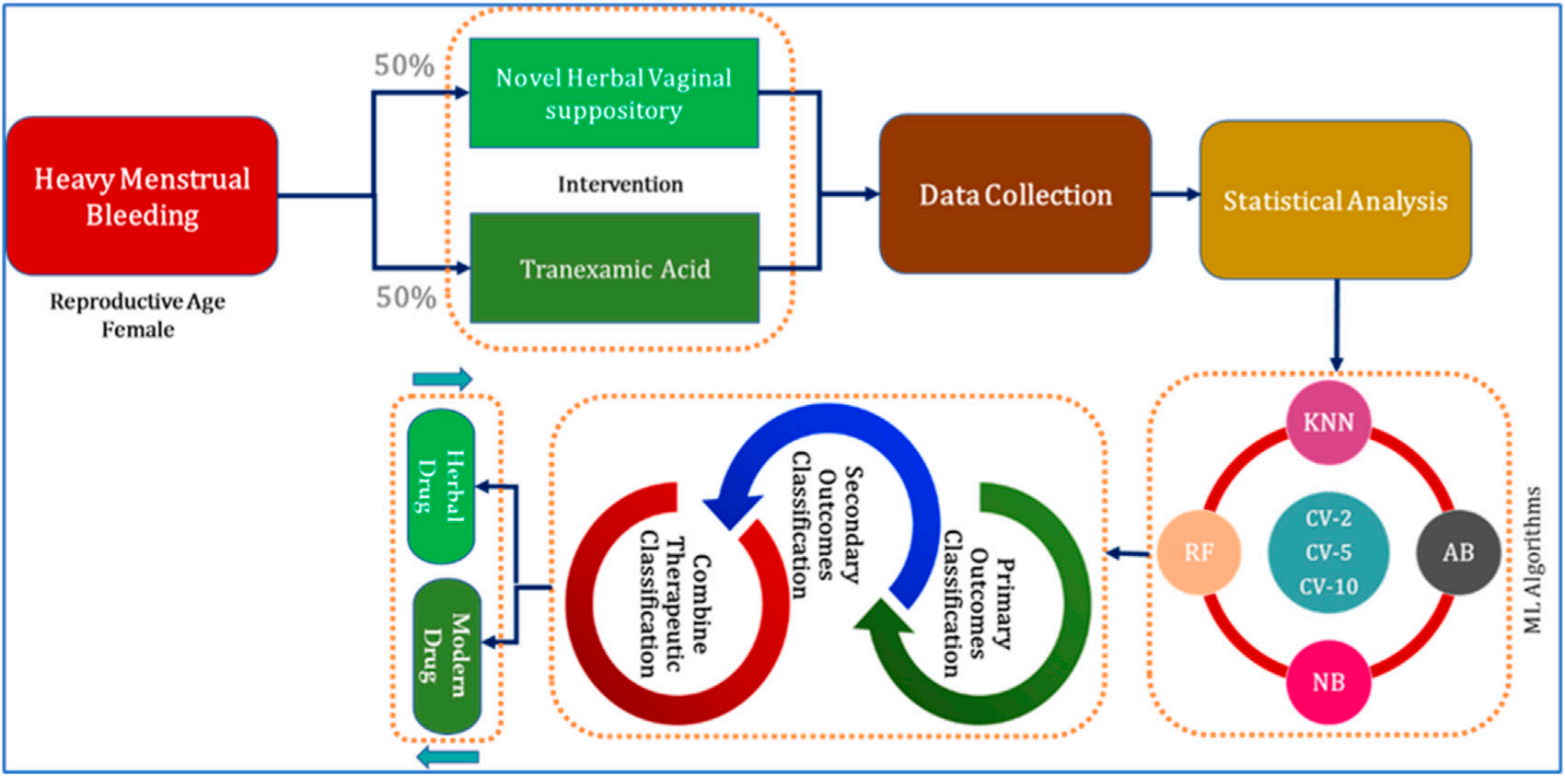
Organizational diagram of the present study.

This study included stages such as protocol designing, approval from the scientific review committee, ethical approval, clinical trial registry, participant enrollment, data collection, and analysis of data results (Figure 1).

### 2.1 Ethical statement

The protocol was completed in line with the principles of the “*Declaration of Helsinki”* (2013) and the “*GCP guidelines of the Ministry of AYUSH.”* This study was approved by the institutional review and ethics committees. Informed consent was obtained from all randomized participants.

### 2.2 Experimental design and data collection

The study followed the design of a single-center, randomized, double-dummy, standard-controlled study with two parallel arms (1:1) in the National Institute of Unani Medicine, Bengaluru. Married women with regular cycles (21–35 days) within the reproductive age group of 18–45 years and complaints of HMB for more than 7 days, an amount of flow >80 mL, or both (Yousefi et al., 2020) were included. Participants were excluded if they had pelvic pathology (uterine fibroids >3 cm and/or >3 in number, polyp, and PID) and malignancy. Diagnosed cases of bleeding disorders, severe anemia (Hb < 7 g%), chronic renal disease, endocrine disorders (uncontrolled thyroid dysfunction), liver disease, uncontrolled hypertension, and diabetes mellitus, along with individuals with a history of using hormonal contraceptives in the last 3 months, pregnant women, and lactating mothers, were also excluded. Throughout the study, every participant had the choice to discontinue at any point.

We collected the demographic, clinical, and behavioral characteristic data during the recruitment interview of both the suppository and tranexamic groups. Kuppuswamy’s scale of 2020 was used for assessing socioeconomic status (Saleem and January 2021). A general physical examination, bimanual examination, and PBLAC score for HMB were recorded. Furthermore, laboratory investigations such as hemogram, random blood sugar, clotting time, bleeding time, platelet count, thyroid-stimulating hormone, and abdominopelvic ultrasound sonography (USG) were conducted at baseline to exclude general diseases and pelvic diseases. During the trial period, the participants were instructed to use the barrier method for contraception and refrain from using any other medicines during the menstrual phase. Participants included in the study were asked to report any treatment-related adverse effects.

### 2.3 Intervention protocol

#### 2.3.1 Selection of novel vaginal suppository

After reviewing the literature, the formulation of acacia gum (*Gond Babul*) and camphor (*Kafoor*) vaginal suppository was selected for the management of HMB as they possess astringent, hemostatic, and anti-inflammatory properties (Kabir al-Din, 2007; Saeedi et al., 2020). Furthermore, scientific studies on Acacia gum reported its powerful astringent, anti-inflammatory, antimicrobial, styptic, and analgesic properties (Saeedi et al., 2020). Camphor, a terpenoid with the chemical formula C_10_H_16_O, is obtained by distillation of the wood of *C. camphora* and has been traditionally employed to address various symptoms, including inflammation, infection, congestion, and muscle pain (Lee et al., 2022). Camphor is proven to have anti-inflammatory, analgesic, antioxidant, and astringent properties (Karashima et al., 2007; Fan et al., 2020; Hussain et al., 2021; Lee et al., 2022). Acacia gum was confirmed and verified through authentication procedures. It was labeled as *Acacia arabica* (Lam.) Willd. [Fabaceae] with an authentication number FRLHT No. 5575. Camphor was identified and authenticated in the Regional Research Institute of Unani Medicine, Chennai, India, with no. DSRU/DTL No.09, 10/2022-23. In addition, the plant’s scientific names were cross-checked in “Kew Plants of the World Online” and “World Flora Online.” For further reference, the specimens of camphor and *A. arabica* gum were submitted to our institute’s pharmacology department with voucher specimen number 104/UQ/Res/2021.

#### 2.3.2 Standardization of the vaginal suppository drugs

The qualitative and quantitative analysis of vaginal suppositories was carried out at the “*Regional Research Institute of Unani Medicine, Chennai*.” Microbial load testing for the vaginal suppository encompassed assessments for total fungal count, total bacterial count, Enterobacteriaceae, *E. coli, Salmonella* spp., and *S. aureus*. Results indicated that the total bacterial and fungal counts in the vaginal suppository sample were less than 1 cfu/g, well within permissible limits. Furthermore, the samples were found to be free from microbes such as *Salmonella* spp., *E. coli,* Enterobacteriaceae, and *S. aureus*. In addition, heavy metal analysis, encompassing lead, cadmium, arsenic, and mercury, was conducted. The analysis of the vaginal suppository revealed the absence of lead, cadmium, arsenic, and mercury.

The qualitative densitometry HPTLC analysis of the vaginal suppository was carried out at the Drug Safety Research Unit at the Regional Research Institute of Unani Medicine, Chennai, India. One gram of the vaginal suppository was separately dissolved in a mobile phase prepared with toluene:ethyl acetate:methanol (9.0:0.7:0.3). Solvent systems were sampled in various volume ratios, *viz.*, toluene:ethyl acetate (7:3), toluene:ethyl acetate (9:1), toluene:ethyl acetate:methanol (7:2:1), toluene:ethyl acetate:ethyl formate:formic acid (6:2:1:1), and toluene:ethyl acetate:methanol (9:0.7:0.3). The most appropriate solvent system was toluene:ethyl acetate:methanol 9:0.7:0.3 (v/v/v).

Analysis was completed on 10 × 10 cm silica gel 60 F254 plates. The sample solution, Linomat 5 (CAMAG, Switzerland), an automated spray-on band applicator equipped with a 100-µL Hamilton syringe, was used and operated with the following settings: a band width of 8 mm, distance from the plate edge of 12.5 mm, migration of 8 cm, and distance from the bottom of the plate of 10 mm. The test sample solution (5 μL) was applied on tracks A and B for vaginal suppositories, respectively, on the TLC plate using the HPTLC ATS4 system. Development of the plates was carried out after allowing a twin trough chamber (CAMAG, Switzerland) for saturation at room temperature for 20 min. After development, the plates were allowed to air-dry at room temperature. Subsequently, fingerprints and densitometric chromatograms were recorded under UV 254 nm and 366 nm and visible light, and after derivatization with anisaldehyde sulfuric acid, the plates were kept in an oven at 110°C. The process was monitored using the CAMAG TLC Visualizer and scanned using the CAMAG TLC Scanner 3. The R_f_ values of the spots were calculated as follows: R_f_ = distance traveled by the spot/distance traveled by the solvent front (Sethi, 1996).

A significant color variation in HPTLC of vaginal suppository was observed on tracks A and B under UV 254. The spot with Rf values of 0.70 and 0.92 is light black. It was also observed that on tracks A and B under UV 366, a lack of spots appeared in the absorbance mode. However, in the case of fluorescence mode track A under UV 366, three spots with R_f_ values of 0.34 (light blue), 0.98 (light blue), and 0.97 (light blue) were observed. In addition, in track B under UV 366, three spots with R_f_ values of 0.38 (light blue), 0.87 (light blue), and 0.98 (light blue) are observed. After derivatization under white light, spots with R_f_ values of 0.45 (dark blue) and 0.55 (light pink) were observed on tracks A and B. The chromatograms of acacia gum and vaginal suppository at UV 254 and 366 nm revealed that all sample constituents were separated without any tailing or diffuseness.

**FIGURE 2:**
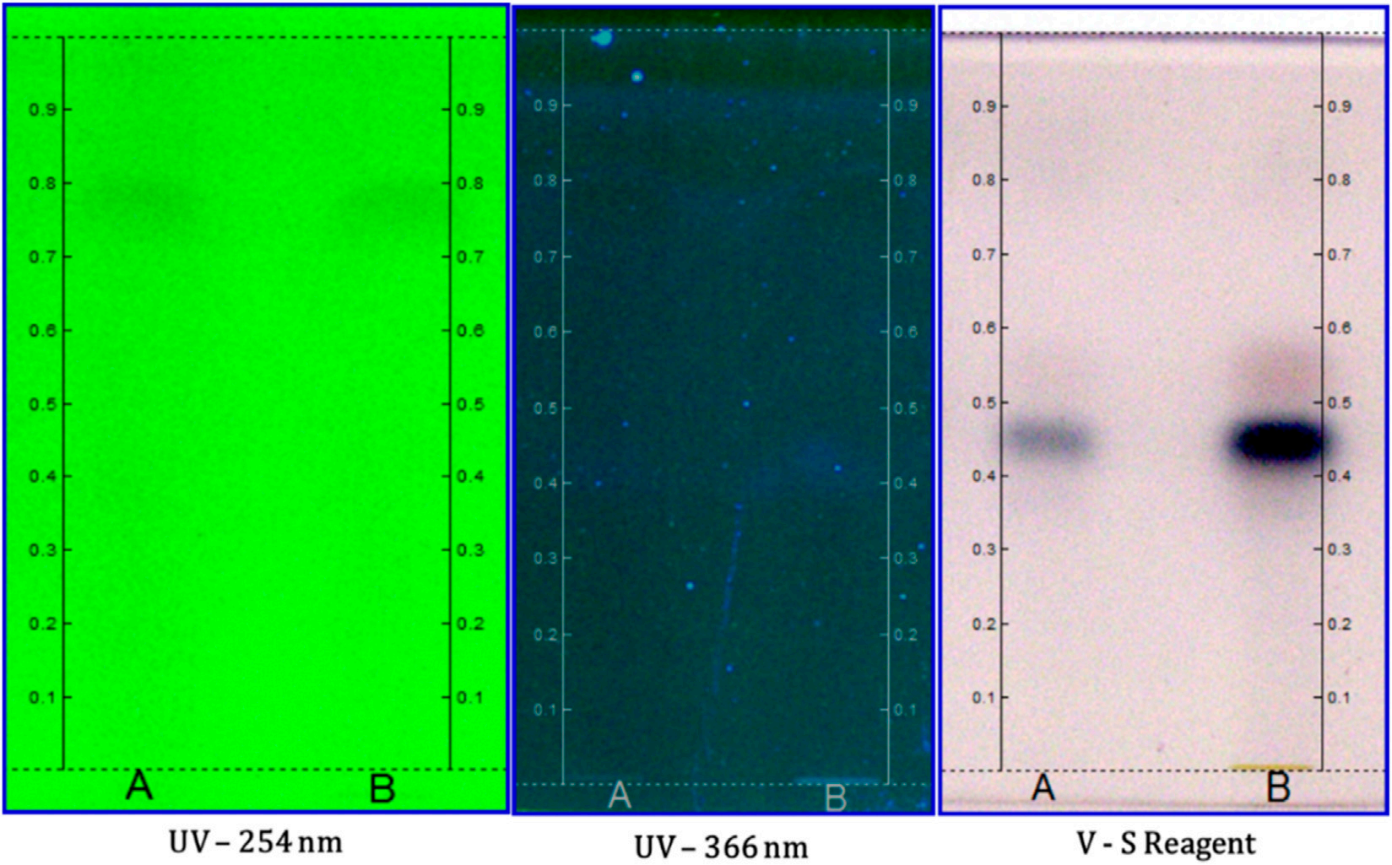
HPTLC fingerprint analysis of the suppository with tracks A and B at different wavelengths.

In the HPTLC fingerprinting analysis, various peaks were detected, and it was found that some metabolites with various colors under the UV wavelength appeared in track B in comparison to track A. Thus, the developed chromatogram will be specific to the selected solvent system: toluene:ethyl acetate:methanol (9:0.7:0.3), providing an R_f_ value, and serves as a better tool for the standardization of the drug (Figure 2).

#### 2.3.3 Preparation of the vaginal suppository

**FIGURE 3:**
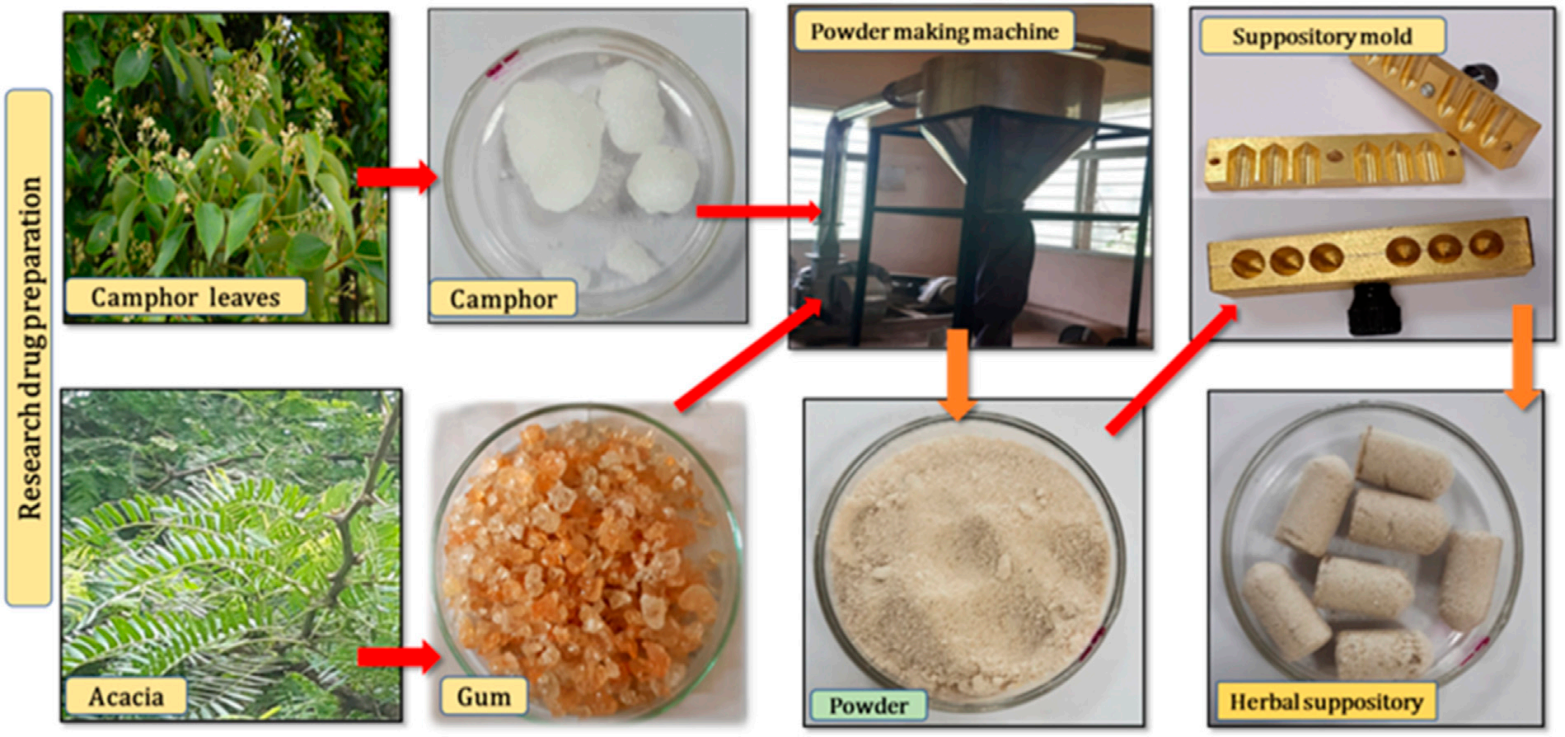
Preparation of the novel vaginal suppository of Acacia gum (*Gond Babul*) and Camphor (*Kafoor*).

The vaginal suppository was made at the National Institute of Unani Medicine, India (Figure 3). Acacia gum and camphor crude plant materials were purchased from the local market and were cleaned. After cleaning, the medicine was finely powdered separately in the powder-making machine. The powder was passed through a sieve, with a mesh size of 80. Then, the fine powder of both plant materials was thoroughly mixed. The mixture of camphor and acacia gum was tightly filled in a brass suppository mold with six holes and sealed to make a vaginal suppository. Cellulose powder (500 mg) was filled in capsules (size 0) using a capsule filling machine.

#### 2.3.4 Dosage of the SG and TG

During the initial 5 days of the menstrual cycle, participants in the SG received one oral placebo capsule twice a day. Simultaneously, from the first day to the seventh day of the menstrual cycle, two vaginal suppositories (each weighing 3,500 mg) were administered per vaginum at bedtime for three consecutive cycles. On the other hand, participants in the TG were administered the standard drug, tranexamic acid (500 mg), orally twice a day from day one to day five of the menstrual cycle. Simultaneously, from day one to day seven of the menstrual cycle, two placebo vaginal suppositories made of palm sugar were administered per vaginum at bedtime for three consecutive cycles.

### 2.4 Pictorial blood loss assessment chart for HMB

The first PBLAC, created in 1990 by Higham et al. (1990), featured three graphics (icons) that represented different brands of feminine products that had been progressively stained with blood (Magnay et al., 2020). Towels received 1, 5, and 20 points on the icon scale, whereas tampons received 1, 5, and 10 points. Different blood quantities resulted in visually comparable stain sizes. As a result, the score given to each icon was based on the applied blood volume in mL; however, it was not always equal to that amount. Although there were no linked scores, the diameter of the currency was compared to the size of the blood clot, and the number of flooding incidents was noted (Magnay et al., 2020). The PBLAC’s specificity and sensitivity for detecting menorrhagia are 80% and 88%, respectively (Yousefi et al., 2020). During each follow-up, a change in the PBLAC mean score was determined. Additionally, calculations were performed for PBLAC scores categorized as either <100 or >100. Participants with a PBLAC score of less than 100 were considered to have normal menstrual bleeding. A score surpassing 100 was regarded as indicative of heavy menstrual bleeding.

All participants were evaluated at baseline (BL) and treatment follow-up (TF1, TF2, and TF3) every month for three successive menstrual cycles after menstruation to assess the PBLAC score and adverse events. A follow-up without treatment (FF1) was conducted after menstruation in the fifth cycle to determine the sustained effect of vaginal suppository and tranexamic acid.

### 2.5 SF-36 HRQoL questionnaire and hemoglobin (HB %) estimation

During each follow-up, a change in the SF-36 HRQoL score was determined. The total score and eight health-related dimensions that contribute to the scores were assessed at the baseline and post-intervention (TF3). To further categorize the SF-36 dimensions, the physical component summary (PCS) for physical functioning and the mental component summary (MCS) for emotional wellbeing were also assessed in both groups at the baseline and post-intervention (Qaraaty et al., 2014). All participants were evaluated at baseline and treatment follow-up (TF3) after menstruation to assess the SF-36 score and hemoglobin levels.

Hemoglobin estimation was carried out at baseline and post-intervention to observe the improvement in HB% post-intervention from baseline.

### 2.6 Assessment of the safety of the SG and TG

Medication safety was assessed by clinical history, physical examination, measuring vital signs, and monitoring adverse events at each follow-up.

### 2.7 Assessment of hematological and safety biochemical markers

Hematological and safety biochemical markers were measured at baseline in the SG and TG. At the pre-screening visit, blood was collected from recruited participants and was permitted to clot for 15–30 min at room temperature. For serum preparation, the clotted blood sample was centrifuged at 3,000–3,500 rpm for 5–10 min. Biochemical marker evaluation was performed with kits purchased from BioSystems, manufactured by BioSystems Diagnostics Pvt. Ltd., India, using the BA 200 LED Technology Automatic Biochemistry Analyzer. The glucose oxidase/peroxidase method was used to measure glucose (Trinder, 1969). Alanine aminotransferase (ALT) and aspartate aminotransferase (AST) levels were measured using the IFCC method (Schumann et al., 2010). The alkaline phosphatase (ALP) level was predicted using the 2-amino-2-methyl-1-propanol buffer (IFCC) (Tietz et al., 2011). The urease/glutamate dehydrogenase method was used to estimate blood urea levels (Gutman, n.d.). S. creatinine levels were assessed using Jaffe’s method (M Peake, 2006).

### 2.8 Sample size estimation

For an outcome variable on the SF-36 score with a minimum difference of 52 post-treatment in a two-group randomized study with a 5% level of significance and 90% statistical power, the sample size of 62 (31 in each group) was estimated with a 10% attrition rate (Goshtasebi et al., 2015).

### 2.9 Randomization and allocation concealment

The second investigator, using randomization software, generated a random list. The randomization process employed a simple random sampling method, creating an open list of random numbers within a single block. The allocation ratio was 1:1, and the randomization order was kept confidential from the initial investigator until each patient was allocated to receive an intervention.

### 2.10 Masking and blinding

The participants were blinded using a process that included masking and matching, achieved by administering the medication in a non-transparent sachet to both groups. A double-dummy technique was also employed to maintain blinding in a trial, as the interventions being compared had different routes of administration. In this technique, the SG received placebo oral capsules and a per-vaginal research suppository, while the TG was given oral standard tranexamic acid capsules and a placebo suppository per-vaginal. However, the double-dummy technique adds complexity to the trial design and involves more intensive monitoring to ensure compliance and assess outcomes accurately. Despite this challenge, this technique was adopted to maintain blinding, as it is crucial for the validity of study results. The analyst conducting the study was also blinded and unaware of the group assignments.

### 2.11 Treatment adherence

Every month, participants were given research and standard control drugs in a pouch until their next planned visit. They were trained to return all unused medicine at each visit. To determine the number of medicines taken, the remaining suppository and tablets/capsules were counted and subtracted from the number provided. In addition, to enhance compliance, each participant was called on their mobile at the time of menstruation to take their medication.

### 2.12 Statistical methods

The statistical program SPSS version 28.0.0 was used. The mean (standard deviation) of the quantitative variables was displayed. The alpha error was established at 0.05 with a 95% confidence level; a two-sided *p*-value was employed; and 80% of the study’s power was achieved. In view of the design of this study, the Mann–Whitney U test was used to compare the effect of vaginal suppositories vs tranexamic acid on primary and secondary efficacy parameters. For intragroup and inter-group comparison, repeated measures of ANOVA were analyzed for follow-up data of each group. All efficacy parameters were analyzed as per intention-to-treat principles using data from all randomized participants who have taken treatment for at least one menstrual cycle. The last observation carried forward (LOCF) approach was used to impute missing data.

### 2.13 Machine learning techniques

In the current study, we used four types of machine learning algorithms, namely, KNN, AB, NB, and RF, to analyze the experimental research data (AlShorman et al., 2022; Sultana et al., 2022a; Iqbal et al., 2022; Ullah et al., 2022).

#### 2.13.1 K-nearest neighbor classifier

The KNN classifier is an extensively used machine learning algorithm employed for both classification and regression tasks, particularly valuable in scenarios where data exhibit intricate and non-linear relationships. The underlying principle of the KNN classifier revolves around the concept of similarity. When tasked with classifying a new data point, the algorithm identifies the ‘k’ closest neighbors from the training dataset, where ‘k’ is a user-defined parameter. The selection of neighbors is determined by a chosen distance metric, often the Euclidean or Manhattan distance, which quantifies the dissimilarity between data points based on their feature values. During the training phase, KNN stores the entire training dataset, retaining its respective class labels for reference (Lai et al., 2019).

In the prediction phase, KNN calculates the distances between the new data point and every point in the training dataset. The ‘k’ data points with the smallest distances to the new point are chosen as neighbors. The class label for the new data point is then determined by a majority vote among these ‘k’ neighbors. This flexible approach allows KNN to adapt to varying data distributions and decision boundaries, making it effective at capturing complex patterns in the data. However, the algorithm does come with certain considerations. For instance, the choice of ‘k’ can significantly impact the model’s performance, influencing its sensitivity to noise and overfitting. Additionally, KNN can be computationally expensive, particularly when dealing with large datasets, and requires careful preprocessing steps such as feature scaling to ensure that no single feature dominates the distance calculations.

#### 2.13.2 AdaBoost classifier

The AdaBoost classifier is a prominent ensemble learning algorithm for its remarkable ability to enhance the accuracy of weak learners and improve overall predictive performance. Introduced by Yoav Freund and Robert Schapire in the mid-1990s, AdaBoost addresses the task of combining multiple weak learners—individual models that perform slightly better than random chance—to create a single, robust model with strong predictive capabilities. At its core, AdaBoost functions through an iterative process that emphasizes the misclassified instances in each successive round. During each iteration, the algorithm assigns higher weights to misclassified data points, compelling subsequent weak learners to focus on those difficult examples. This adaptive weight assignment effectively directs the attention of the algorithm toward the instances that present the greatest challenge. Consequently, the ensemble model evolves to excel at handling complex decision boundaries and capturing intricate data patterns (Pal et al., 2023).

Throughout the iterations, AdaBoost trains a sequence of weak learners, often simple decision trees or stumps, and progressively combines their outputs to create an ensemble prediction. Importantly, each weak learner contributes to the final prediction with a weight proportional to its accuracy, ensuring that the most reliable models hold the most influence. AdaBoost’s strengths lie not only in its capacity to amplify the performance of modest models but also in its relative insensitivity to overfitting. It avoids the pitfalls of overfitting by focusing on difficult instances, which helps prevent the algorithm from becoming overly tailored to the training data. While AdaBoost does exhibit exceptional performance in many scenarios, it may be sensitive to noisy data and outliers, potentially leading to undue emphasis on misclassified instances and affecting overall accuracy.

#### 2.13.3 Naive Bayes classifier

The naive Bayes classifier is a machine learning algorithm renowned for its simplicity and efficiency in handling classification tasks. Rooted in Bayesian probability theory, naive Bayes operates to make predictions. Despite its “naive” assumption of feature independence, which may not hold in all cases, naive Bayes often yields impressive results and serves as a strong baseline model (AlShorman et al., 2022).

The algorithm’s working principle involves estimating the probability of a given class label for a new data instance based on the joint probabilities of its features. Naive Bayes makes use of Bayes’ theorem to compute these probabilities. In the prediction phase, the algorithm applies Bayes’ theorem to compute the posterior probabilities for each class and assigns the class label with the highest probability to the new data instance.

#### 2.13.4 Random forest classifier

The random forest classifier stands as a cornerstone in the realm of ensemble learning, offering a robust and versatile approach to classification tasks. Rooted in the concept of decision tree ensembles, random forest addresses the limitations of individual trees by combining the predictive power of multiple trees, thereby mitigating issues of overfitting and enhancing overall accuracy. This algorithm, introduced by Leo Breiman and Adele Cutler, operates by constructing a multitude of decision trees during both the training and prediction phases (Heyat et al., 2019).

In a random forest, each tree is built using a bootstrapped subset of the original training data, ensuring diversity among the trees. Moreover, during the tree-building process, a random subset of features is selected for each split, promoting different paths of decision making and reducing the likelihood of a single dominant feature dictating the outcome. Once all trees are constructed, the algorithm aggregates their predictions through a majority vote (for classification tasks) or an average (for regression tasks) to arrive at a final prediction. This ensemble approach lends itself to improved generalization and robustness.

## 3 Results

**FIGURE 4:**
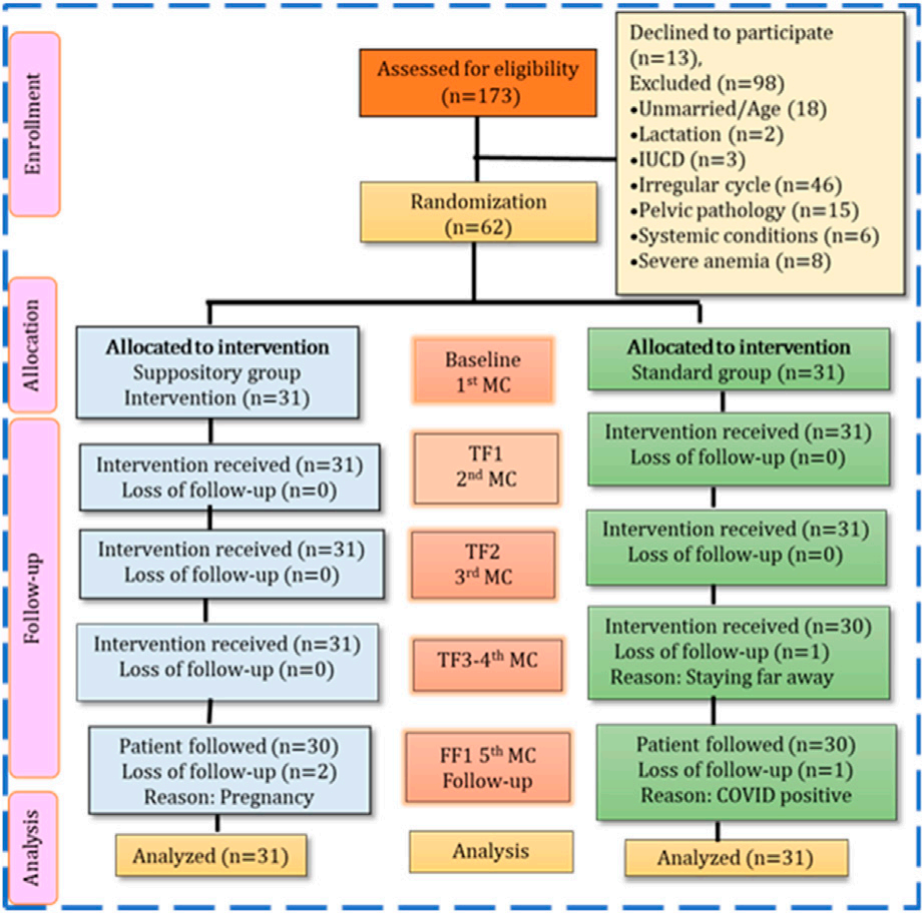
Study flow of the participants based on consort statement.

The study duration was from 2 February 2021 to 17 February 2022. In this study, a total of 173 participants with HMB were screened according to inclusion criteria. Ninety-eight participants were omitted from the trial for different reasons, and 13 participants declined to participate. The remaining 62 participants were randomly assigned to the study. The randomly assigned 62 participants were divided into two groups: the suppository group and tranexamic acid group (standard control). The loss to follow-up is summarized in Figure 4.

### 3.1 Baseline sociodemographic, clinical, and temperament parameters

Statistical tests demonstrated the insignificant difference between the SG and TG (*p* > 0.05) in the baseline sociodemographic parameters in terms of age, religion, habitat, duration of illness, diet, BMI, and socioeconomic characteristics. The mean age in the SG and TG was 35.61 ± 6.27 and 35.61 ± 5.54 years, respectively. A significant proportion of participants were identified as Muslim, with 74.19% in the SG and 83.87% in the TG. Additionally, the majority of participants in both groups belonged to the upper-lower socioeconomic status, comprising 74.1% in the SG and 70.9% in the TG. The details can be found in Table 1.

**TABLE 1:** Statistical analysis of the suppository and tranexamic acid groups based on baseline sociodemographic parameters.

Parameter	Suppository group (n = 31)	Tranexamic group (n = 31)	Total (n = 62)	*p-*value
Age (years)				
20–29	6 (19.35)	6 (19.35)	12 (19.35)	0.83^a^
30–39	16 (51.61)	18 (58.06)	34 (54.83)	
40–45	9 (29.03)	7 (22.58)	16 (25.80)	
Religion no. (%)				
Muslim	23 (74.19)	26 (83.87)	49 (79.03)	0.53^a^
Hindu	8 (25.8)	5 (16.12)	13 (20.96)	
**Habitat**				0.70^a^
Urban	28 (90.32)	26 (83.87)	54 (87.10)	
Rural	3 (9.68)	5 (16.12)	8 (12.90)	
**Duration of illness (months)**	19 ± 4.40	14.12 ± 2.75	-	0.35^b^
Diet				
Vegetarian	4 (12.90)	3 (9.6)	7 (11.29)	0.99^a^
Non-vegetarian	18 (58.06)	20 (64.51)	38 (61.29)	
Mixed	9 (29.03)	8 (25.80)	17 (27.42)	
**BMI (Kg/m** ^**2**^ **)**	26.92 ± 4.66	28.43 ± 4.89	-	0.21^b^
Socioeconomic status				
Upper I	0	0	0	0.44^c^
Upper middle II	1 (3.23)	2 (6.25)	3 (4.84)	
Lower middle III	5 (16.13)	7 (22.58)	12 (19.35)	
Upper lower IV	23 (74.19)	22 (70.97)	45 (72.58)	
Lower V	2 (6.25)	0	2 (3.22)	

^a^Chi-square test

^b^ Unpaired *t*-test.

^c^ Fisher exact test; data are presented as the mean ± SD or no. %.

### 3.2 Efficacy parameters

#### 3.2.1 Primary outcome results

In the SG, the mean PBLAC score at baseline was 635.322 ± 504.23. Subsequently, at post-intervention (TF3) and the fifth cycle (FF1), it decreased to 67.70 ± 22.37 and 70.51 ± 33.23, respectively, demonstrating a statistically extremely significant reduction from the baseline (*p* < 0.0001). In the TG, the PBLAC score was 512.93 ± 283.57 at baseline, and it notably decreased to 97.96 ± 39.25 at post-intervention (TF3) and 104.64 ± 47.81 during the fifth cycle (FF1), indicating a statistically extremely significant reduction from the baseline (*p* < 0.0001) (Table 2 (a)). A higher percentage of participants in the SG achieved normal menstrual blood loss compared to the TG (93.5% vs 74.2%) at post-intervention (TF3) (Table 2 (b)). A statistically significant reduction in the mean PBLAC score was observed at each follow-up in the SG compared to the TG (*p* < 0.001). Table 3 provides a summary of the cycle duration as well as the duration and amount of menstrual blood flow in both the SG and TG.

**TABLE 2:** PBLAC score of both groups.

(a) PBLAC mean score at each follow-up in both groups							
Follow-up	Suppository group (n = 31)	95% CI (L-U)	Difference from BL	Tranexamic group (n = 31)	95% CI (L-U)	Difference from BL	*p*-value
**BL**	635.322 ± 504.23	450.40–820.25	-	512.93 ± 283.57	408.94–616.93	-	0.50^a^
**TF1**	96.93 ± 52.98***	77.50–116.37	538.39	144.87 ± 67.73***	120.03–169.71	368.06	0.002**^a^
**TF2**	75.74 ± 29.08***	65.07–86.41	559.58	110.709 ± 45.55***	94.00–127.42	402.23	0.0009***^a^
**TF3**	67.70 ± 22.37***	59.50–75.91	567.61	97.96 ± 39.25***	83.56–112.37	414.97	0.0018**^a^
**FF1**	70.51 ± 33.23***	58.32–87.70	564.81	104.64 ± 47.81**	87.10–122.18	408.29	0.0003***^a^

(b) Participants with normal PBLAC scores (<100) and HMB (>100) in both groups					
Follow-up	Suppository group (n = 31)			Tranexamic group (n = 31)	
	<100 (normal menstrual bleeding)	>100 (heavy menstrual bleeding)		<100 (normal menstrual bleeding)	>100 (heavy menstrual bleeding)
**BL**	0	31 (100)		0	31 (100)
**TF1**	19 (61.3)	12 (38.7)		10 (32.3)	21 (67.7)
**TF2**	27 (87.1)	4 (12.9)		19 (61.3)	12 (38.7)
**TF3**	29 (93.5)	2 (6.5)		23 (74.2)	8 (25.8)
**FF1**	29 (93.5)	2 (6.5)		20 (64.5)	11 (35.5)

^a^ Mann–Whitney U test; **p< 0.001 is considered strongly significant; ***p< 0.0001 is considered extremely significant from baseline; **BL,** baseline; **TF1, TF2, and TF3**, treatment follow-up months; **FF1,** follow-up without treatment; **PBLAC,** pictorial blood lost assessment chart. Data are presented as the mean ± SD and no. %.

**TABLE 3:** Statistical analysis of both groups based on different variables.

Variable	Suppository group (n = 31)	95% CI (L-U)	Difference from BL	Tranexamic group (n = 31)	95% CI (L-U)	Difference from BL	*p*-value
Duration of the cycle							
BL	28.54 ± 3.1	27.40–29.68		28.45 ± 1.52	27.89–29.01		0.70^a^
TF3	28.87 ± 2.34	28.96–2.3	−0.33	29.19 ± 2.33	27.58–29.44	−0.74	0.90^a^
*p*-value	0.49			0.85			
Overall *p*-value between the groups = 0.24^b^							
Duration of flow							
BL	10.8 ± 5.17	8.43–12.14		8.67 ± 3.9	7.23–10.11		0.07^a^
TF1	5.44 ± 1.55***	8.33–6.00	5.36	5.51 ± 1.56***	4.94–6.09	3.16	0.76^a^
TF2	5.16 ± 1.44***	4.79–6.18	5.64	5.51 ± 1.56***	4.91–6.09	3.16	0.16^a^
TF3	5.16 ± 1.31***	4.68–5.63	5.64	5.16 ± 1.09***	5.09–5.93	3.51	0.62^a^
FF1	5.36 ± 1.44***	4.61–5.70	5.44	5.22 ± 1.25***	4.75–5.56	3.45	0.96^a^
Overall *p*-value between the groups = 0.04^b^							
Amount of flow (no. of pads/cycle)							
BL	34.16 ± 25.49	24.81–43.51		27.45 ± 14.84	22.00–32.89		0.35^a^
TF1	8.64 ± 2.8***	7.60–9.68	25.52	9.54 ± 4.30***	7.97–11.12	17.91	0.69^a^
TF2	7.74 ± 2.12***	6.96–8.52	26.42	8.83 ± 3.23***	7.65–10.02	18.62	0.29^a^
TF3	7.48 ± 1.69***	6.86–8.10	26.68	8.09 ± 2.05***	7.34–8.85	19.36	0.20^c^
FF1	7.70 ± 1.9***	6.98–8.43	26.46	8.41 ± 2.95***	7.33–9.50	19.04	0.27^c^

^a^ Mann–Whitney U test.

^b^ RMANOVA.

^c^ Unpaired *t*-test. ***p< 0.0001 is considered extremely significant from baseline; BL, baseline; TF, treatment follow-up; FF, first follow-up without treatment.

#### 3.2.2 Secondary outcome results

A significant improvement in the SF-36 HRQoL score in the SG compared to the TG was noted in the sub-score of the physical health composite score (*p* = 0.01), mental health component score (*p* = 0.01), and total SF-36 score (*p* = 0.02) at post-intervention (TF3). Furthermore, significant improvement in physical function (*p* = 0.04), general health (*p* = 0.01), the role of limitation of the emotional problem (*p* = 0.04), and social function (*p* = 0.02) in the SG was noted in comparison with the TG at post-intervention. The intragroup comparison of both groups at post-intervention from baseline showed extremely significant improvement (*p* < 0.0001) in all parameters of the SF-36 score (Table 4). At post-intervention (TF3), the hemoglobin level was increased in 11 participants (35.48%) in the SG and 10 participants (32.25%) in the TG, with a significant difference (*p* < 0.05) (Table 5).

**TABLE 4:** SF-36 HRQoL health survey using statistical techniques in secondary outcomes.

Domain	Period	Suppository group (n = 31)	Tranexamic group (n = 31)	*p*-value
Physical function (PF)	Baseline	68.70 ± 19.53	69.51 ± 18.04	0.99^a^
	TF3	88.22 ± 12.14	79.35 ± 20.19	0.04^a^
	*p-*value	<0.0001^b^	<0.001^b^	
Role limitation due to physical problems (RF)	Baseline	37.09 ± 43.71	34.35 ± 38.46	0.96^a^
	TF3	81.45 ± 33.52	72.25 ± 35.8	0.28^a^
	*p-*value	<0.0001^b^	<0.0001^b^	
Bodily pain (BP)	Baseline	48.7 ± 17.11	45.64 ± 13.52	0.96^a^
	TF3	67.82 ± 14.54	59.75 ± 12.84	0.28^a^
	*p-*value	<0.0001^c^	<0.0001^b^	
General health (GH)	Baseline	38.96 ± 9.65	39.63 ± 9.32	0.78^d^
	TF3	62.14 ± 11.78	55.64 ± 10.89	0.01^a^
	*p-*value	<0.0001^c^	<0.0001^b^	
Role limitation due to emotional problems	Baseline	54.83 ± 39.95	47.30 ± 42.83	0.48^a^
	TF3	96.77 ± 10.02	73.11 ± 38.89	0.04^a^
	*p-*value	<0.0001^b^	<0.0001^b^	
Energy/fatigue/vitality (VT)	Baseline	48.83 ± 11.27	48.38 ± 12.40	0.71^a^
	TF3	63.70 ± 10.56	63.70 ± 11.75	0.9^d^
	*p*-value	<0.0001^b^	<0.0001^c^	
Emotional wellbeing (mental Health-MH)	Baseline	53.58 ± 11.36	54.70 ± 11.28	0.69^d^
	TF3	63.83 ± 10.25	64.58 ± 9.5	0.92^a^
	*p-*value	0.0001^c^	0.0001^c^	
Social functioning (SF)	Baseline	48.95 ± 14.60	47.98 ± 12.11	0.85^a^
	TF3	64.51 ± 12.54	56.85 ± 9.5	0.02^a^
	*p*-value	<0.0001^b^	0.0002^b^	
Physical health composite score (PCS)	Baseline	48.36 ± 17.43	47.28 ± 14.77	0.79^d^
	TF3	74.91 ± 14.93	66.75 ± 16.44	0.01^a^
	*p*-value	<0.0001^c^	<0.0001^b^	
Mental health composite score (MCS)	Baseline	51.15 ± 15.30	49.59 ± 16.53	0.70^d^
	TF3	72.20 ± 8.26	64.56 ± 15.55	0.01^d^
	*p*-value	<0.0001^c^	<0.0001^b^	
Total score	Baseline	49.76 ± 15.57	48.44 ± 14.51	0.73^d^
	TF3	73.56 ± 11.09	65.65 ± 14.81	0.02^d^
	*p-*value	<0.0001^c^	<0.0001^c^	

**TF3**: Post-intervention (third treatment follow-up).

^a^Mann–Whitney U test.

^b^ Wilcoxon matched pair test.

^c^ Paired Student’s ‘t’ test.

^d^ Unpaired Student’s ‘t’ test; TF, treatment follow-up.

**TABLE 5:** Hematological and safety biochemical markers of the SG and TG.

Variable (mean ± SD)	Suppository group (n = 31)	Tranexamic group (n = 31)	*p-*value
Hemoglobin (g/dL)			
Baseline	11.06 ± 1.36	10.6 ± 1.38	0.21^a^
Fourth cycle (TF3)	11.01 ± 1.33	10.79 ± 1.21	0.49^a^
*p-*value	0.26^b^	0.39^b^	
Coagulation profile			
Platelet count	3.17 ± 0.60	3.35 ± 0.70	0.30^a^
Lakhs/cu mm			
Bleeding time (min)	2.05 ± 0.19	2.13 ± 0.34	0.61^c^
Clotting time (min)	5.44 ± 0.46	5.34 ± 0.43	0.43^c^
Random blood sugar (mg/dL)	100.48 ± 19.48	98.88 ± 14.80	0.80^c^
Thyroid-stimulating hormone (µIU/mL)	2.68 ± 1.10	2.55 ± 1.30	0.65^a^
Safety profile			
Alanine aminotransferase (IU/L)	22.93 ± 7.5	24.67 ± 9.59	0.76^c^
Aspartate aminotransferase (IU/L)	31.54 ± 8.3	32.51 ± 10.69	0.69^a^
Alkaline phosphatase (IU/L)	100.35 ± 19.83	112.38 ± 35.29	0.10^a^
Blood urea (mg/dL)	20.70 ± 5.56	21.29 ± 5.45	0.67^c^
Serum creatinine (mg/dL)	0.87 ± 0.13	1.05 ± 1.11	0.95^c^
Ultrasonography of the abdomen			
USG abdomen no. (%)			
Normal 1)	19 (61.29)	21 (67.74)	0.89^d^
Bulky 2)	2 (6.45)	2 (6.45)	
Fibroid 3)	3 (9.67)	1 (3.25)	
Adenomyosis 4)	3 (9.67)	3 (9.67)	
Mixed 5)	4 (12.90)	4 (12.90)	

^a^ Unpaired Student’s ‘t’.

^b^ Paired student’s ‘t’ test.

^c^ Mann–Whitney U test.

^d^ Chi-square test.

### 3.3 Hematological, coagulation profile, safety biochemical markers, and ultrasonography of the abdomen in the SG and TG

All parameters (thyroid-stimulating hormone, clotting time, bleeding time, and platelet count) were within the normal limit at the baseline in the suppository and tranexamic acid groups with an insignificant difference (*p* > 0.05) (Table 5). Hepatic (AST, ALT, and ALP) and renal (blood urea and S. creatinine) safety parameters were within normal limits at baseline.

### 3.4 Classification (SG vs. TG) results using machine learning algorithms

#### 3.4.1 Primary outcome classification

The current study employed a combination of three distinct cross-validation (CV) strategies: CV-2, CV-5, and CV-10, in primary outcome classification (SG vs. TG) analysis (Table 6). Each model was evaluated across various performance metrics, including AUC, accuracy, precision, recall, and specificity. In the context of the CV-2 model, the AB classifier emerged as the top performer, exhibiting exceptional results across all measures: AUC (57.90%), accuracy (57.80%), precision (58.20%), recall (57.80%), and specificity (60.00%). Within the CV-5 framework, the KNN classifier took the lead, achieving the highest scores for AUC (52.10%), accuracy (57.10%), precision (57.00%), recall (57.10%), and specificity (58.50%). Remarkably, in the CV-10 configuration, the AB classifier excelled with remarkable prowess, yielding superior performance metrics, including AUC (58.60%), accuracy (60.30%), precision (60.00%), recall (60.30%), and specificity (61.60%).

**TABLE 6:** Primary outcome classification (SG vs. TG) based on different machine learning models.

Classifier	Model	AUC	Accuracy	Precision	Recall	MCC	Specificity
KNN	CV-2	0.484	0.460	0.453	0.460	−0.064	0.477
AB		0.579	0.578	0.582	0.578	0.191	0.600
NB		0.454	0.254	0.503	0.254	−0.007	0.746
RF		0.518	0.524	0.532	0.524	0.075	0.539
KNN	CV-5	0.521	0.571	0.570	0.571	0.162	0.585
AB		0.466	0.476	0.469	0.476	−0.032	0.493
NB		0.487	0.079	0.375	0.079	−0.082	0.864
RF		0.558	0.556	0.552	0.556	0.129	0.569
KNN	**CV-10**	0.436	0.429	0.414	0.429	−0.134	0.446
**AB**		0.586	**0.603**	0.600	0.603	0.224	0.616
NB		0.470	0.048	0.246	0.048	−0.066	0.924
RF		0.495	0.524	0.515	0.524	0.063	0.539
Mean		0.504	0.425	0.484	0.425	0.038	0.616
±SD		0.046	0.184	0.097	0.185	0.113	0.144

Bold values mean highest value.

**FIGURE 5:**
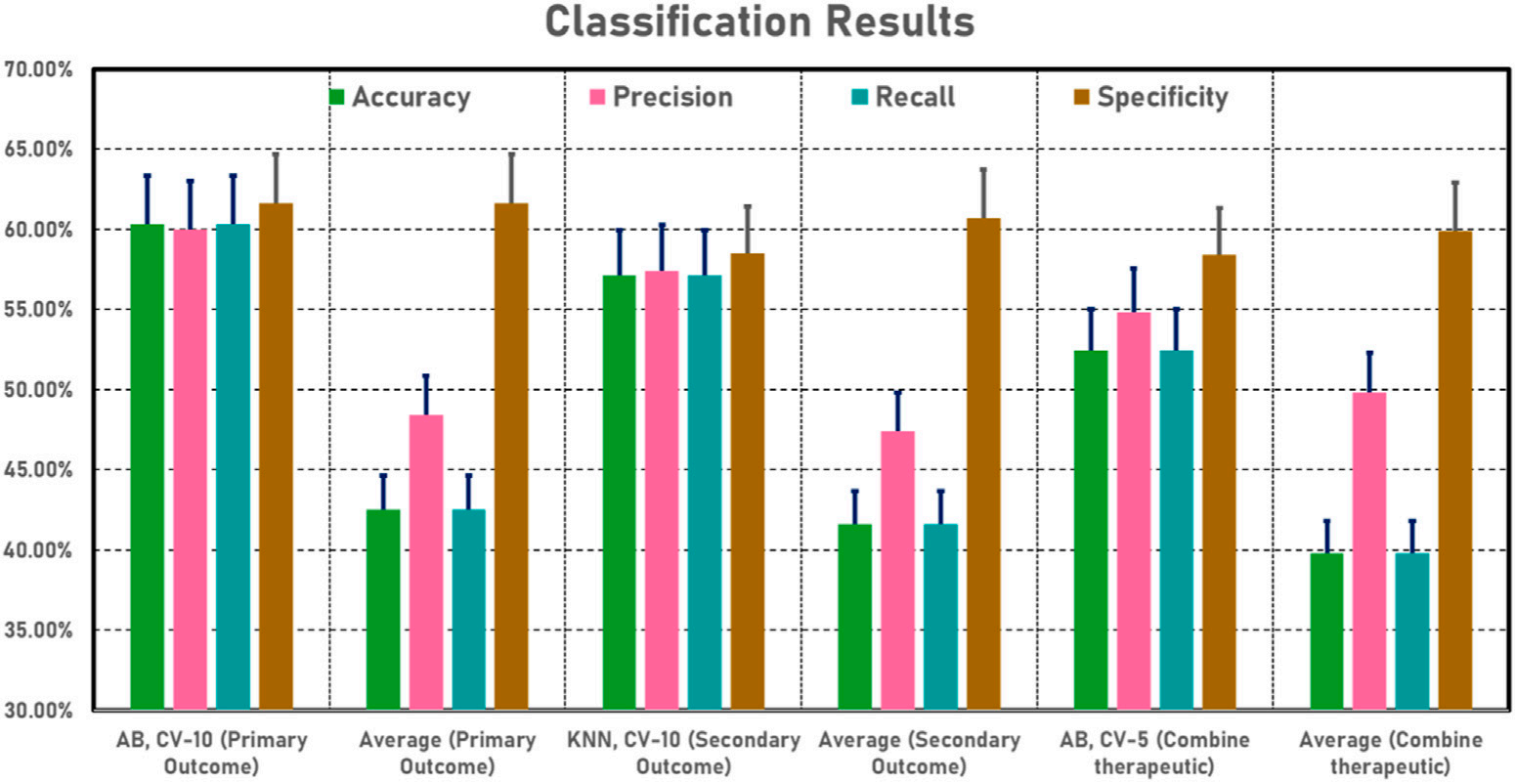
Classification (SG vs. TG) results based on machine learning models.

Upon examining the collective outcomes, the AB classifier demonstrated its prowess by achieving the highest accuracy (60.30%) within the CV-10 model, showcasing its efficacy in this specific setting (Figure 5). Conversely, the NB classifier, operating within the CV-10 model, exhibited the lowest accuracy (4.80%), underlining its limitations within this context. This in-depth exploration of classification results elucidates the varying capabilities of machine learning models under different cross-validation scenarios and provides insights into their strengths and weaknesses in accurately categorizing the given data.

#### 3.4.2 Secondary outcome classification

The current study employed a combination of three distinct CV strategies: CV-2, CV-5, and CV-10, in secondary outcome classification (SG vs. TG) analysis (Table 7). Each model was evaluated across various performance metrics, including AUC, accuracy, precision, recall, and specificity. In the context of the CV-2 model, the AB classifier emerged as the top performer, exhibiting exceptional results across all measures: AUC (53.40%), accuracy (54.00%), precision (53.30%), recall (54.00%), and specificity (55.40%). Within the CV-5 framework, the RF classifier took the lead, achieving the highest scores for AUC (60.40%), accuracy (57.10%), precision (56.20%), recall (57.10%), and specificity (58.50%). Remarkably, in the CV-10 configuration, the KNN classifier excelled with remarkable prowess, yielding superior performance metrics, including AUC (59.10%), accuracy (57.10%), precision (57.40%), recall (57.10%), and specificity (58.50%).

**TABLE 7:** Secondary outcome classification (SG vs. TG) based on different machine learning models.

Classifier	Model	AUC	Accuracy	Precision	Recall	MCC	Specificity
KNN	CV-2	0.538	0.476	0.469	0.476	−0.031	0.493
AB		0.534	0.540	0.533	0.540	0.095	0.554
NB		0.512	0.270	0.514	0.270	0.022	0.761
RF		0.475	0.444	0.434	0.444	−0.095	0.462
KNN	**CV-5**	0.563	0.508	0.502	0.508	0.032	0.523
AB		0.421	0.429	0.421	0.429	−0.125	0.446
NB		0.490	0.095	0.410	0.095	−0.045	0.880
**RF**		0.604	**0.571**	0.562	0.571	0.157	0.585
**KNN**	**CV-10**	**0.591**	**0.571**	**0.574**	**0.571**	**0.166**	**0.585**
AB		0.526	0.540	0.531	0.540	0.094	0.554
NB		0.487	0.048	0.246	0.048	−0.066	0.924
RF		0.550	0.508	0.503	0.508	0.032	0.523
Mean		0.524	0.416	0.474	0.416	0.019	0.607
±SD		0.049	0.173	0.085	0.173	0.091	0.152

Bold values mean highest value.

Upon examining the collective outcomes, the KNN classifier demonstrated its prowess by achieving the highest accuracy (57.10%) within the CV-10 model, showcasing its efficacy in this specific setting (Figure 5). Conversely, the NB classifier, operating within the CV-10 model, exhibited the lowest accuracy (4.80%), underlining its limitations within this context. This in-depth exploration of classification results elucidates the varying capabilities of machine learning models under different cross-validation scenarios and provides insights into their strengths and weaknesses in accurately categorizing the given data.

#### 3.4.3 Combine therapeutic classification

In current research, comprehensive combined therapeutic classification (TG vs. SG) analysis employed a combination of three distinct CV strategies: CV-2, CV-5, and CV-10 (Table 8). Each model was evaluated across various performance metrics, including AUC, accuracy, precision, recall, and specificity. In the context of the CV-2 model, the RF classifier emerged as the top performer, exhibiting exceptional results across all measures: AUC (47.60%), accuracy (49.20%), precision (48.40%), recall (49.20%), and specificity (50.80%). Within the CV-5 framework, the AB classifier took the lead, achieving the highest scores for AUC (53.20%), accuracy (52.40%), precision (54.80%), recall (52.40%), and specificity (58.40%). Notably, in the CV-10 configuration, the KNN classifier excelled with remarkable prowess, yielding superior performance metrics, including AUC (55.80%), accuracy (52.40%), precision (52.00%), recall (52.40%), and specificity (53.90%).

**TABLE 8:** Combined therapeutic (SG vs. TG) classification based on different machine learning models.

Classifier	Model	AUC	Accuracy	Precision	Recall	MCC	Specificity
KNN	CV-2	0.489	0.460	0.452	0.460	−0.066	0.477
AB		0.468	0.429	0.457	0.429	−0.047	0.522
NB		0.490	0.238	0.451	0.238	−0.035	0.731
RF		0.476	0.492	0.484	0.492	0.000	0.508
KNN	**CV-5**	0.534	0.492	0.486	0.492	0.000	0.508
**AB**		**0.532**	**0.524**	**0.548**	**0.524**	**0.104**	**0.584**
NB		0.473	0.127	0.609	0.127	0.030	0.910
RF		0.530	0.508	0.499	0.508	0.032	0.523
**KNN**	**CV-10**	0.558	**0.524**	0.520	0.524	0.066	0.539
AB		0.401	0.413	0.404	0.413	−0.160	0.431
NB		0.473	0.063	0.574	0.063	−0.016	0.940
RF		0.526	0.508	0.500	0.508	0.031	0.523
Mean		0.495	0.398	0.498	0.398	−0.005	0.599
±SD		0.040	0.155	0.054	0.155	0.065	0.161

Bold values mean highest value.

Upon examining the collective outcomes, the AB classifier demonstrated its prowess by achieving the highest accuracy (52.40%) within the CV-5 model, showcasing its efficacy in this specific setting (Figure 5). Conversely, the NB classifier, operating within the CV-10 model, exhibited the lowest accuracy (6.30%), underlining its limitations within this context. This in-depth exploration of classification results elucidates the varying capabilities of machine learning models under different cross-validation scenarios and provides insights into their strengths and weaknesses in accurately categorizing the given data.

## 4 Discussion

The present study found that the PBLAC score was meaningfully reduced in the SG and TG from the baseline measurement at each follow-up, showing a reduction in HMB. At post-intervention (TF3), 29 participants (93.5%) in the test group and 23 participants (74.2%) in the tranexamic group had normal MBL (less than 80 mL), showing that the SG was more effective than the TG. In the second (TF1), third (TF2), fourth (TF3), and fifth (FF1) cycles, PBLAC scores decreased significantly in the SG compared to the TG. In the SG and TG, the mean PBLAC score decreased from 635.322 ± 504.23 to 67.70 ± 22.37 and 512.93 ± 283.57 to 97.96 ± 39.25, respectively, at post-intervention (TF3), demonstrating a statistically significant difference (*p* < 0.001). A higher percentage of participants in the SG achieved normal menstrual blood loss compared to the TG (93.5% vs 74.2%). The SG showed a considerable improvement in total SF-36 scores (73.56%) compared to the TG (65.65%), with a statistically significant difference (*p* < 0.001). Additionally, no serious adverse events were reported in either group. Notably, machine learning algorithms, particularly AB and KNN, demonstrated the highest accuracy within cross-validation models for both primary and secondary outcomes. There was an insignificant difference in the duration of the cycle (*p* = 0.240) between the SG and TG. Moreover, participants receiving suppositories reported statistically clinically significant improvements in HRQoL compared to those receiving tranexamic acid.

A noteworthy reduction in the PBLAC score was noted during treatment from baseline to post-intervention follow-up (*p* < 0.001). This finding aligns with the previous studies (Imdad et al., 2017; Azizkhani et al., 2018; Yousefi et al., 2020). According to the work of Goshtasebi et al. (2015), there was an insignificant difference between *Punica granatum* and tranexamic acid on HMB and quality-of-life improvement. Imdad et al. (2017) confirmed that *Qurs Gulnar* was more effective than tranexamic acid for HMB. Yousefi et al. (2020) reported no significant difference between the efficacy of the Golnar tablet with tranexamic acid in HMB. As per the work of Azizkhani et al. (2018), women treated with cupping experienced significantly lower PBLAC scores at one and 3 months compared to those treated with medroxyprogesterone acetate. In the current study, the secondary endpoints were HRQoL and Hb%. It was noticeable that the suppository group showed significant improvement in women’s QoL in all parameters at TF3 and FF1 from baseline. These findings were similar to the previous study (Jahan et al., 2016). At post-intervention (TF3), the Hb% was increased by 35.48% in the SG and 32.25% in the TG, with a significant difference (*p* < 0.05).

### 4.1 Role of inflammation, oxidative stress, and immune responses in uterine bleeding and the possible mechanisms of action of botanical drugs to ameliorate HMB

Intrauterine leukocytes and their molecular by-products play an essential inflammatory role in both normal and pathological uterine hemorrhage. According to current data, immune cell disruption and associated cytokine mediators are causal factors for abnormal uterine bleeding (AUB) and pelvic pain (Berbic et al., 2014). Intrauterine leucocytes and their derivatives play main ‘inflammatory’ roles in normal menstruation and AUB. Menstruation has recently been linked to an increase in tissue leukocyte counts and their pro-inflammatory mediator. They are crucial in endometrial breakdown, proliferation, and remodeling. Endothelial cells secrete various pro-inflammatory cytokines during mitochondrial failure, including IL-6, IL-1, and TNF-a, as well as an elevation of ICAM-1 expression, which attracts monocyte activation and adhesion. During the menstrual cycle in the endometrium, macrophages, NKc, large granular lymphocytes, eosinophils, and mast cells are explicitly increased. In addition, during the proliferative phase following an endometrial breakdown, to expedite clearance of the endometrial cavity, CD8 cytotoxic T-cell activity is enhanced, which shows that this factor supports the concept of an activated adaptive component of the immune system. During normal menstruation, the immune responses seem to be tightly regulated. It is believed that MMP-2 and MMP-9 (matrix metalloproteinase) expression levels interfere with hemorrhage (Berbic et al., 2014). As per another study, compared to normal women, women with HMB have higher serum levels of prostaglandin E2 and prostacyclin, which cause localized vasodilation and platelet accumulation, and lower levels of prostaglandin F2, which cause vasoconstriction and PGE2 receptors (Kashefi et al., 2015). The current evidence emphasizes the importance of mitochondrial function in immune cell activity. During immunological reactions, mitochondrial physiology, morphology, and metabolism are tightly regulated. T-cell activation necessitates the formation of ROS, whereas activated T cells can use both OXPHOS and glycolysis for proliferation (Chen et al., 2018). According to research, foods that cause inflammatory reactions in the body contribute to HMB. In exchange, natural remedies or pharmaceutical drugs that prevent the production of PGs and leukotrienes may have an anti-inflammatory impact and reduce menstrual blood loss (Berbic et al., 2014). ROS accumulation, which causes oxidative stress, has been linked to improper oocyte maturation, decreased fertilization, and the formation of endometriosis. The balance of ROS and antioxidants is critical for the health of women. Mumford et al. (2016) contend that the interactions between serum antioxidants and endogenous hormones are critical for the menstrual cycles of premenopausal women (Mumford et al., 2016).

Unani treatments have the potential to treat HMB as they have astringent, analgesic, and anti-inflammatory properties and avoid hormonal side effects. Unani scholars mention the use of acacia gum to treat hemoptysis and menorrhagia (Yousefi et al., 2020) as it possesses ethnomedicinal activities such as astringent, styptic, and anti-inflammatory (Kabir al-Din, 2007), with specific therapeutic action that is useful in stopping menstrual bleeding (Kabir al-Din, 2007). Additionally, acacia gum possesses pharmacological and therapeutic potential, including anti-inflammatory, analgesic (Fan et al., 2020), prostaglandin inhibiting (Lee et al., 2006), hepatoprotective (Johari et al., 2015), vasoconstrictor (Amos et al., 1999), hemostatic, and astringent properties. Acacia gum has been documented to have plant metabolites such as phenolics, saponins, alkaloids, flavonoids, tannins (gallic acid, ellagic acid, and tannic acid), vitamin C, stearic acid, Arabin, carotene, crude protein, carbohydrates crude fiber, calcium, magnesium, and selenium (Ali, 2012). Tannins, gallic acid, and other flavonoids are considered to have strong astringent and styptic activities and, thus, can cause the contraction of the capillary endothelium (Tansaz et al., 2016). The astringent 0 exerts an impact on the biosynthesis of prostaglandins and reduces uterine bleeding (Livdans-Forret et al., 2007). The calcium in the acacia gum helps maintain the hemostatic mechanism (Nadkarni KM, 2004). Vitamin C with bioflavonoids helps decrease HMB by making the capillaries stronger and also preventing their fragility (Livdans-Forret et al., 2007). Camphor possesses ethnomedicinal properties such as astringent, disinfectant, and neutralizing blood actions due to its cold and dry, anti-inflammatory (Khan and Muhammad, 2018), and externally anesthetic and analgesic properties (Kabir al-Din, 2007). Camphor research studies show that it possesses pharmacological and therapeutic potential such as anti-inflammatory (Liu et al., 2020), analgesic (Fan et al., 2020), hepatoprotective, antioxidant (Muhamad et al., 2019), estrogenic (Maerkel et al., 2007), and prostaglandin inhibiting properties (Liu et al., 2020). Camphor has phenols, flavonoids (tannins), saponins, alkaloids, and carbohydrates. The effect of camphor on estrogenic gene expression was studied (Maerkel et al., 2007). According to their findings, 4-MBC administration in rats had sex- and region-specific effects on the mRNA levels of PPE, ER-alpha, PR, and IGF-I (Maerkel et al., 2007). A variety of liver illnesses have been treated using camphor as a hepatoprotective drug.

Tannin, a plant secondary metabolite, has analgesic, hemostatic, and anti-inflammatory properties. Flavonoids have anti-inflammatory, antioxidant, and analgesic properties (Safari et al., 2016). Flavonoids can scavenge lipid peroxyl, hydroxyl, and superoxide anion radicals, and they play an important role in the prevention of illnesses caused by oxidative damage to membranes, proteins, and DNA. Alkaloids and saponins possess anti-inflammatory properties. Anti-inflammatory activity is aided by antioxidant properties (Abdulhamid et al., 2019). Polyphenols, which are natural plant metabolites found in plants, have a wide range of biological activities. Before cell viability is seriously compromised, phenolic plant metabolites and flavonoids can interact with ROS/RNS to stop the chain reaction (Taghvaei and Jafari, 2015). Several authors have established a link between inflammation and oxidative stress. Evidence suggests that oxidative stress is pathogenic in chronic inflammatory diseases (Hussain et al., 2016). Antioxidants have anti-inflammatory actions that limit nociceptor activity and reduce the production and/or release of prostaglandins, which act as inflammatory pain mediators. By blocking the NF-kB pathway, a substance can exhibit both antioxidant and anti-inflammatory characteristics (Agnieszka and Skrzydlewska, 2022). A study mentioned that the production of the inflammatory cytokines TNF-α and IL-6 can be reduced by taking vitamin C (an antioxidant) orally. When given intravenously, it can help prevent cytokine storms. *C. camphora* contains cineol, borneol, and citronellal, which inhibit inflammatory cytokine, chemokine production, and PGE-2 production (Fazmiya et al., 2022). *C. camphora* has potent anti-inflammatory (Kang et al., 2019) and antioxidant properties (Zafar et al., 2012). Eucalyptol, camphor, and linalool were extracted, and nine terpenoids were obtained from the essential oil of *C*. *camphora* (Wu et al., 2020). These inhibit the production of TNF-α, IL-6, and PGE2 and improve the increase of mRNA and protein levels of iNOS, COX-2, and MMP-9 in LPS-stimulated RAW 264.7 macrophages (Li et al., 2018). *C. camphora* has anti-inflammatory mechanisms that limit the synthesis of NO and PGE2 in LPS/IFN-activated macrophages. Its MeOH extract inhibits 70% of the synthesis of PGE2 in LPS/IFN-activated macrophages. Another study found that *C. camphora* has anti-inflammatory properties due to its ability to modulate cytokine production, NO and PGE2 release, functional activation of adhesion molecules, and oxidative stress. Additionally, *C. camphora* can significantly modulate numerous inflammatory responses at the transcriptional level (Lee et al., 2006).

**FIGURE 6:**
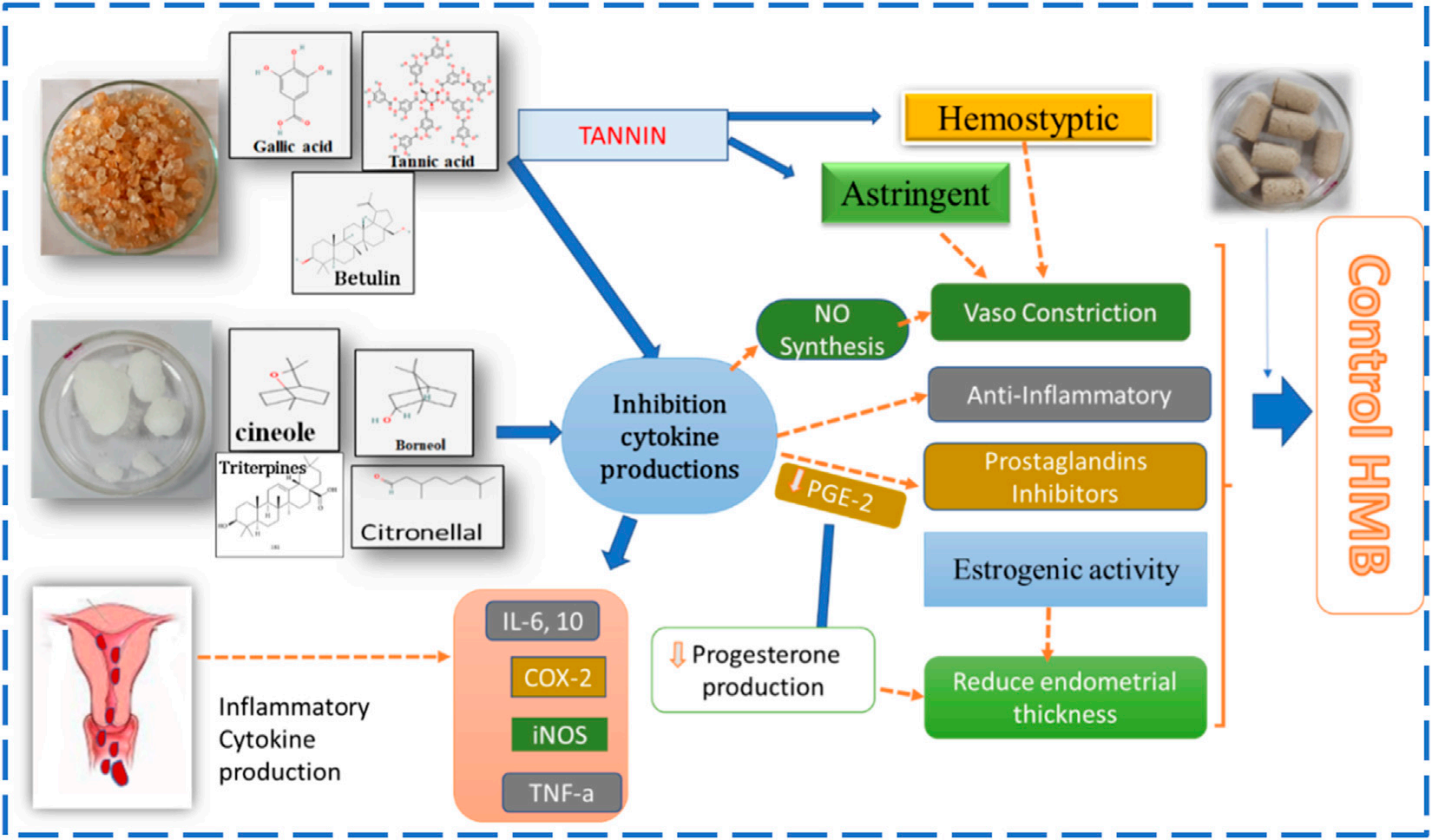
Anti-inflammatory mechanism of action of a vaginal suppository of camphor and acacia gum.

**FIGURE 7:**
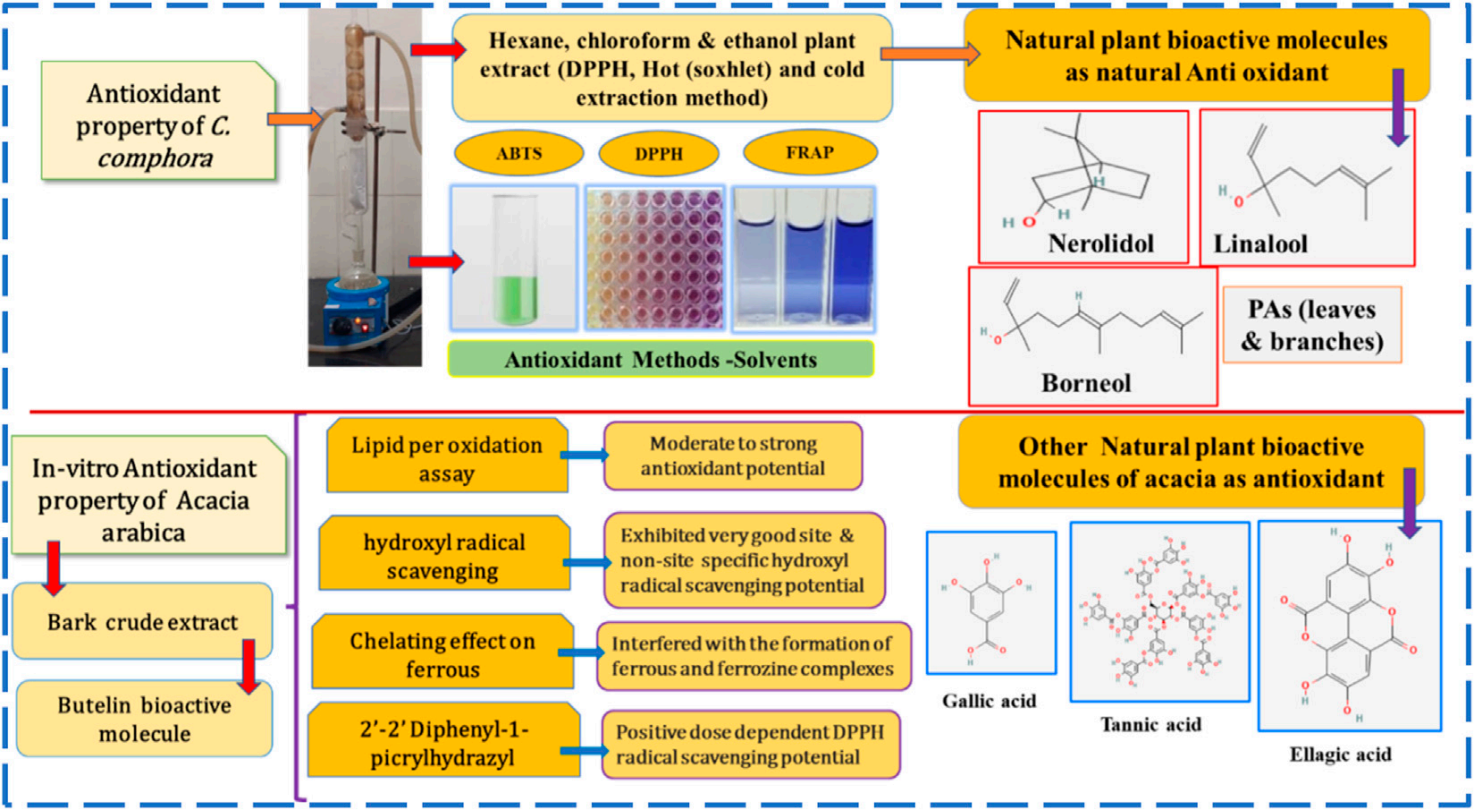
Antioxidant activity of camphor and acacia.

Oxidative stress has been linked to several diseases’ pathogenesis, including cardiovascular disease, renal disease, atherosclerosis, hypertension, premenstrual syndrome, and aging (Sultana et al., 2022b). The PAs of *C. camphora* also showed strong antioxidant capacity with the scavenging of DPPH, FRAP, and ABTS assays (Yang et al., 2021). Liu et al. (2019) confirmed that flavonoids extracted from *C. camphora* have antioxidant capacity (Fu et al., 2016). From *C. camphora* ethanolic extract, phenolic plant metabolites, including linalool, nerolidol, and borneol, were extracted (Muhamad et al., 2019) that have antioxidant and remove free radical potential (Fazmiya et al., 2022). Kaddam et al. demonstrated the unique effect of acacia gum as an antioxidant among sickle cell anemic patients, as it increases TAC levels and decreases oxidative stress markers. In addition, acacia gum has immune-modulatory and anti-inflammatory effects. It controls immunity in mice by reducing TNFα and CRP while increasing the IL-10 anti-inflammatory cytokine. It exerts local anti-inflammatory effects by modifying NF-*κ*B in the small intestine (Ali et al., 2020). Kaur et al. (2022) reported the anti-inflammatory and antioxidant activities of the bioactive molecule betulin isolated from *Acacia arabica* bark. Betulin exhibited moderate-to-strong antioxidant potential in a lipid peroxidation assay and was a selective inhibitor of COX-2 (Kaur et al., 2022). Flavonoids and other phenolic plant metabolites are known to target cyclooxygenase-mediated inflammation. A study found that acacia gum has protective and antioxidant effects on the blood, liver, kidney, and cardiovascular system in experimentally induced injuries to these organs and tissues (Hassanien et al., 2019). Methanolic crude extracts (MCEs) of acacia gum demonstrated anti-inflammatory activity at 300 mg/kg, comparable to indomethacin, which suppresses PG synthesis while also inhibiting the initiatory inflammation process, in which histamine, serotonin, and kinin are the primary mediators. Flavonoids, alkaloids, β-carotene, phenolic acids, and tannins are the phytochemical constituents of Acacia gum. Furthermore, acacia gum has higher antioxidant activity than standard gallic acid and has a strong relationship with total phenolic content (AM Elnour et al., 2018). The mechanism of the suppositories as an anti-inflammatory agent is described in Figure 6, and Figure 7 depicts the antioxidant mechanism of camphor and acacia.

Synchronized prothrombotic and antithrombotic reactions involve a coagulation system, platelets, fibrinolysis system, and vessel wall, which maintain the hemostatic balance in the body. However, in the event of oxidative stress, inflammation, and an altered blood flow state inside the vessel, this balance shifts toward prothrombotic, which is exhibited by impaired platelet and leukocyte adhesion, impaired synthesis of PGI2 and NO, and disturbed fibrinolysis (Marcińczyk et al., 2022). Fibrinolysis is a physiologic factor of hemostasis that contributes to the formation of border clots. Nonetheless, excessive fibrinolysis following tissue injury caused by ischemia and reperfusion, trauma, or surgery may contribute to coagulopathy, bleeding, and inflammatory responses (Levy et al., 2018). The thrombin–thrombomodulin complex, when activated, leads to the activation of thrombin fibrinolysis inhibitor and removes lysine residues on fibrin, removing binding sites for plasminogen, and could play an important role in modifiable cross-talk between inflammation and coagulation. After massive trauma, surgery, or ischemia, the ability to locally regulate fibrinolysis is lost, leading to the development of coagulopathy as plasmin generation and subsequent fibrinolysis become systemic. This is the rationale behind the pharmacologic use of tranexamic acid in fibrinolysis disruption (Levy et al., 2018). VEGF-A is dramatically reduced in HMB participants who experience excessive menstrual bleeding (Akhtar et al., 2022). By activating tissue factors, VEGF-A contributes to neovascularization and may induce coagulation pathways. VEGF-A is produced by immune cell types in the endometrium, including macrophages. Therefore, decreased levels could cause extended heavy bleeding patterns and impair endometrial healing in affected women (Berbic et al., 2014). The fibrinolytic system is demonstrated to be over-activated in HMB-affected women during the menstrual stage of their cycle (Maybin and Critchley, 2016). Increased fibrinolysis causes more blood to be lost during endometrial shedding (Maybin and Critchley, 2016). Anti-fibrinolytic drugs reduce subsequent fibrin breakdown by preventing the interaction between plasmin and lysine residues, which slows down the dissolution of clots. Anti-fibrinolytic agents reversibly inhibit lysine binding sites on plasminogen and displace plasminogen from fibrin, and plasmin is prevented from interacting with lysine residues on the fibrin polymer. To alleviate severe menstrual bleeding, anti-fibrinolytic drugs have been suggested (Ac et al., 2018). As per the reports, the anti-fibrinolytic drug tranexamic acid reduces menstrual blood loss by approximately 50% (Maybin and Critchley, 2016). Hemostatic agents can act through platelet aggregation, vasoconstriction, clotting factor activation, or anti-fibrinolytic activity. Bioactive plant metabolites frequently involved in bleeding control are saponins, tannins, glycosides, and other phenolic compounds (Ebrahimi et al., 2020). Plant metabolites and tannins, since antiquity, have been used for their ethnomedicinal properties in traditional medicine. Tannins have been shown in studies to have the potential to prevent thromboembolic events and fibrinolysis systems. Furthermore, tannins have been shown in studies to have profibrinolytic and Edi antifibrinolytic effects. Tannins can influence platelet and endothelial activity (Marcińczyk et al., 2022). Acacia gum is rich in tannin, with the bioactive molecules ellagic acid, gallic acid, and tannic acid. According to a study, the polymeric metabolites of *A. arabica* found in the aqueous extracts reduce the PT and aPTT in mice; these plant metabolites exhibit hemostatic effects and hasten blood coagulation properties (Bhatnagar et al., 2013). A study has shown that *A. arabica* gum, along with *Moringa oleifera* pods, showed thrombogenic activity as the weight of the clots was significantly higher than that of tranexamic acid (Bhatnagar et al., 2013). *A. arabica* might probably have thrombotic action and comparatively fewer side effects than hormonal treatment. Therefore, acacia gum and camphor have multidimensional potent prostaglandin inhibitors, antioxidant, immunomodulatory, astringent, styptic, and anti-inflammatory properties (Liu et al., 2020). Hence, the therapeutic effect of the vaginal suppository of acacia gum and camphor on HMB is explained in comparison with tranexamic acid.

### 4.2 Adverse effects on the SG and TG

Medication safety was assessed by clinical history, physical examinations, measuring vital signs, and monitoring for adverse events. In the SG, three participants complained of a mild burning sensation that persisted for a few seconds to 10 min with no other complaints. In the TG, back pain and headache were complaints by three participants, whereas two participants complained about flatulence. Similarly, adverse effects with tranexamic acid were reported in a previous study (Lukes et al., 2010).

### 4.3 Strengths of the study

This study holds the distinction of being the first of its kind to validate the efficacy and safety of a vaginal Unani dosage form in HMB. The trial’s design was robust, employing a double-blind, randomized, double-dummy clinical approach, demonstrating good compliance with a low attrition rate of only 10%.

### 4.4 Limitations of the study

A limitation of the study was the omission of the alkaline hematin method, which is considered the standard for calculating menstrual blood loss. However, this method is deemed impractical, time-consuming, and costly. This study focused exclusively on the reproductive age group experiencing heavy menstrual bleeding. Consequently, its applicability cannot be extended to individuals facing peri-menopausal or pubertal heavy menstrual bleeding. Additionally, the majority of participants belonged to the lower-middle-class socioeconomic bracket, limiting the generalization of findings to the upper socioeconomic status. Furthermore, it is important to note that the study’s scope does not include populations with specific conditions. These conditions include uterine fibroids exceeding 3 cm and/or numbering more than 3, as well as individuals with polyps, pelvic inflammatory disease, bleeding disorders, severe anemia (Hb < 7 gm%), chronic renal disease, uncontrolled thyroid dysfunction, liver disease, uncontrolled hypertension, and diabetes mellitus. Therefore, in this context, further studies are recommended. Another limitation of the study was the inability to conduct a quantitative analysis of specific bioactive secondary plant metabolites, stability testing of the vaginal suppository, and an assessment of the pharmacokinetics of the drug molecule due to constraints in resources and time. Furthermore, hepatic safety parameters (AST, ALT, and ALP) and renal safety parameters (blood urea and serum creatinine) have to be carried out after intervention to verify the safety of research botanical drugs on hepatic and renal functions.

### 4.5 Future recommendation

The use of vaginal suppositories may be recommended for women comfortable with complementary and alternative approaches seeking to avoid potential side effects associated with conventional pharmaceuticals. The authors also suggest conducting a quantitative analysis of the specific bioactive secondary metabolites of the plant using diverse mobile phases and evaluating the stability of the finished product. Moreover, assessing the presence of active constituents in the bloodstream could provide insights into comprehensive pharmacokinetic and pharmacodynamic characteristics. To enhance the research drug’s efficacy and potency, further Phase-III clinical trials with an extended duration and lengthier follow-up are suggested. Additionally, a comparative analysis between the vaginal suppository and nonsteroidal anti-inflammatory drugs or other conventional treatments would provide valuable insights.

## 5 Conclusion

We concluded that participants receiving the novel suppository exhibited significant reductions in PBLAC scores and marked improvements in HRQoL SF-36 scores, with a substantial percentage achieving normal menstrual blood loss. Importantly, no adverse events were reported in either group. *A. arabica* gum (*Gond Babul*) and *C. camphora* (*Kafoor*) suppositories emerge as novel and well-supported options for HMB treatment. Furthermore, this study explored the application of AI for the classification of experimental data through machine learning models.

## Figures and Tables

**Table:** 

**4-MBC**	4-Methyl benzylidene camphor
**ABTS assays**	2,2′-Azinobis-(3-ethylbenzothiazoline-6-sulfonic acid
**aPTT**	Activated partial thromboplastin time
**COX**	Cyclooxygenase
**CRP**	C-reactive protein
**DPPH**	2,2-Diphenyl-1-picrylhydrazyl
**ER-alpha**	Estrogen receptor-alpha
**FRAP**	Fluorescence recovery after photobleaching
**ICAM-1**	Intercellular adhesion molecule-1
**IGF-I**	Insulin-like growth factor-I
**IL**	Interleukin
**iNOS**	Inducible nitric oxide synthase
**LPS**	Lipopolysaccharides
**IFN**	Gamma interferon
**MeOH**	Methanol
**MMP-9**	Matrix metalloproteinase
**NF-kB**	Nuclear factor kappa-light-chain-enhancer of activated B cells
**NO**	Nitric oxide
**OXPHOS**	Oxidative phosphorylation
**PGE2**	Prostaglandin E2
**PGI2**	Prostaglandin I2
**PPE**	Preproenkephalin
**PR**	Progesterone receptor
**PT**	Prothrombin time
**ROS/RNS**	Reactive oxygen species/reactive nitrogen species
**TAC**	Total antioxidant capacity
**TNFα**	Tumor necrotizing factor alpha
**VEGF-A**	Vascular endothelial growth factor-A
**CV**	Cross validation
**AI**	Artificial intelligence
**KNN**	K-nearest neighbor
**AB**	AdaBoost
**NB**	Naive Bayes
**RF**	Random forest
**HMB**	Heavy menstrual bleeding
**SG**	Suppository group
**TG**	Tranexamic group
**HRQoL**	Health-related quality of life

## Data Availability

The raw data supporting the conclusion of this article will be made available by the authors, without undue reservation.
