# Supplementary figures and images for: Efficacy of a vaginal suppository formulation prepared with Acacia arabica (Lam.) Willd. gum and Cinnamomum camphora (L.) J. Presl. in heavy menstrual bleeding analyzed using a machine learning technique
Source: Front Pharmacol. 2024 Feb 12;15:1331622. doi: 10.3389/fphar.2024.1331622 (PMC10894987; doi:10.3389/fphar.2024.1331622)

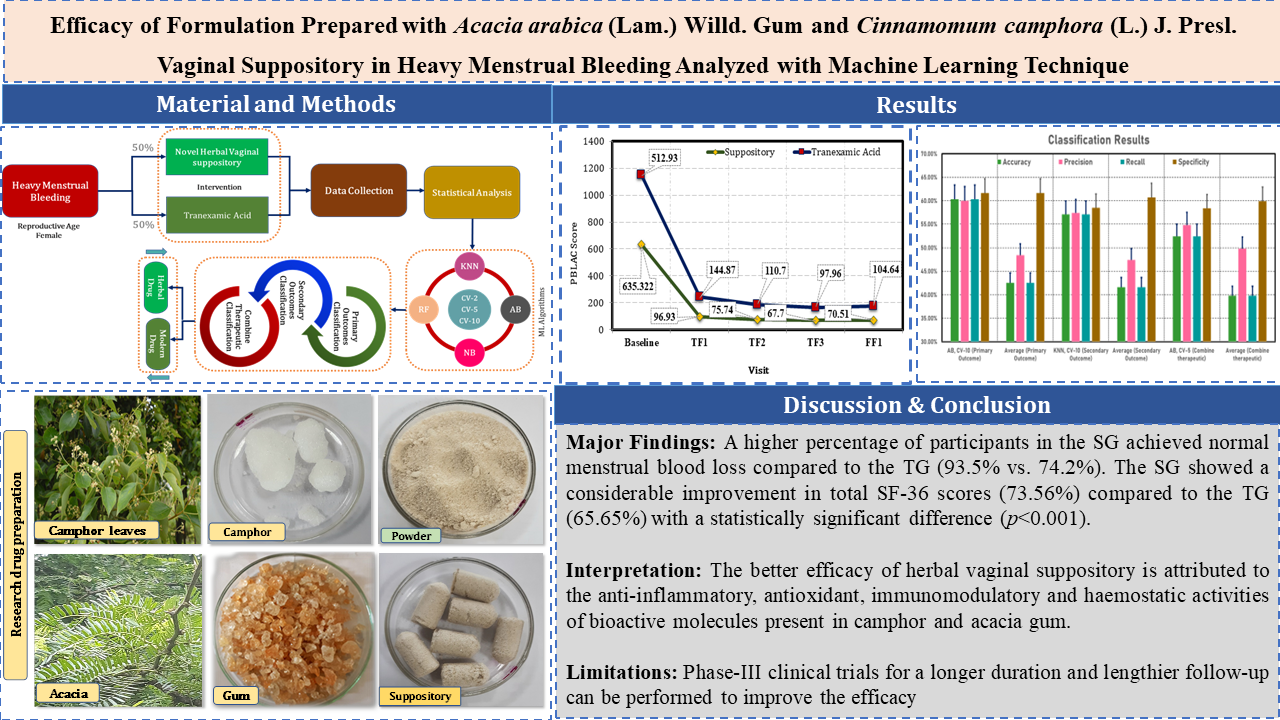

Supplement: Supplementary file 1 [file Image1.PNG]
